# Long-term B-cell depletion with rituximab in relapsing, refractory and severe lupus nephritis: a retrospective case series

**DOI:** 10.1093/ckj/sfag152

**Published:** 2026-05-11

**Authors:** Qiyu Wang, Orhan Efe, Ayman Al Jurdi, Harish Seethapathy, Reza Zonozi, Gabriel Sauvage, Karen Laliberte, John Niles, Anushya Jeyabalan

**Affiliations:** Department of Medicine, Division of Nephrology, Mass General Brigham, Boston, MA, USA; Department of Medicine, Division of Nephrology, Mass General Brigham, Boston, MA, USA; Department of Medicine, Division of Nephrology, Mass General Brigham, Boston, MA, USA; Department of Medicine, Division of Nephrology, Mass General Brigham, Boston, MA, USA; International Kidney Vasculitis and Glomerular Center, Fairfax, VA, USA; Nephrology Associates of Northern Virginia, Fairfax, VA, USA; Department of Medicine, Division of Nephrology, Mass General Brigham, Boston, MA, USA; Department of Medicine, Division of Nephrology, Mass General Brigham, Boston, MA, USA; Department of Medicine, Division of Nephrology, Mass General Brigham, Boston, MA, USA; Department of Medicine, Division of Nephrology, Mass General Brigham, Boston, MA, USA

**Keywords:** B-cell depletion, lupus nephritis, refractory, relapsing, rituximab

## Abstract

**Background:**

B-cell depletion with anti-CD20 agents has been evaluated as part of induction immunosuppression (IS) in lupus nephritis (LN). Data on its long-term efficacy for maintenance of remission remains limited.

**Methods:**

Retrospective case series evaluating outcomes in patients with relapsing, refractory and severe LN at diagnosis who received rituximab (RTX)-induced continuous B-cell depletion for both induction and maintenance at Massachusetts General Hospital from 2008 to 2023.

**Results:**

A total of 26 patients with active LN were included. Eighty-eight percent (23/26) had class III/IV ± V on kidney biopsy; 12% (3/26) had pure class V LN. Median follow-up (from first to last RTX) was 32 months [interquartile range (IQR) 14–68]; median cumulative dose of RTX was 10 g (IQR 6–17). At RTX initiation, all patients received prednisone and oral IS with an intention to taper off oral agents in 12 months. Eighty-one percent (21/26) achieved at least partial remission. Median prednisone dose decreased from 30 to 5 mg/day at 6 months (*P* < .01). Fifty-eight percent (15/26) of patients at 12 months and 79% (11/14) at 24 months were on RTX monotherapy. Six renal relapses occurred in five patients with median time-to-first relapse of 43 months (range 24–142); all episodes but one were preceded by B-cell repopulation. Five patients developed end-stage kidney disease; all had creatinine >2 mg/dL at RTX initiation. Thirty-one percent (8/26) experienced severe infections requiring hospitalization. No deaths occurred.

**Conclusion:**

Long-term continuous B-cell depletion may be an effective treatment strategy in patients with LN who have failed prior IS or with severe disease at diagnosis. Future controlled studies are needed to further evaluate this approach as the backbone therapy in LN.

KEY LEARNING POINTS **What was known:**

- Effective B-cell depletion leads to increase in remission when added to current standard of care therapy in treatment of active lupus nephritis (LN).
- Renal response highly correlates with the duration and depth of B-cell depletion.
- Role of anti-CD20 induced B-cell depletion as long-term maintenance treatment in LN has not been studied.

**This study adds:**

- With rituximab (RTX)-induced continuous B-cell depletion, more than half of the patients with relapsing, refractory or severe nascent LN were able to stop oral immunosuppression and maintain renal remission with RTX monotherapy after 1 year.
- Early B-cell repopulation was more frequent in active LN (∼30% in our cohort) when compared with other autoimmune glomerulonephritis, suggesting that a more intense RTX dosing regimen may be needed to achieve continuous B-cell depletion.
- Hypogammaglobulinemia, chronic inflammatory conditions (bronchitis, vaginitis) and recurrent infections were among the most common adverse effects with prolonged (≥24 months) B-cell depletion.

**Potential impact:**

- In patients with LN who have history of multiple treatment failures and non-adherence, continuous B-cell depletion with RTX may be an option for induction and maintenance of remission.
- Shortening the starting intervals of RTX and guiding maintenance dosing interval with peripheral CD19 monitoring may enhance B-cell depletion and improve early treatment success in patients with active LN.
- Future controlled studies should evaluate long-term continuous B-cell depletion as the backbone of therapeutic approach in LN and extend anti-CD20 agents into the quiescent phase of the disease as maintenance immunosuppression.

## INTRODUCTION

Lupus nephritis (LN) affects nearly 50% of patients with systemic lupus erythematosus (SLE). Up to 30% of patients with class III/IV LN progress to end-stage kidney disease (ESKD) within 15 years [1–5]. Despite advances in LN therapies, nearly half of patients with class III/IV LN fail to achieve remission with current standard of care, and approximately 20% of those who initially responded to immunosuppression (IS) experience relapse within 3–5 years [6–8]. The therapeutic challenges are particularly heightened for patients with relapsing, refractory or severe LN at diagnosis [1, 9].

B cell–targeted therapies are playing an increasingly important role in treatment of LN. Serving as precursors for plasma cells and drivers of clonal expansion of T cells, autoreactive B cells are one of the key components in the pathogenesis of SLE [10]. Despite the initial negative results from the LUNAR (Lupus Nephritis Assessment with Rituximab) trial in 2012, later studies have consistently demonstrated the association between effectiveness of B-cell depletion and renal response [11, 12]. Recent trials on obinutuzumab, a fully humanized anti-CD20 monoclonal antibody which achieves deeper and more sustained B-cell depletion compared with rituximab (RTX), have shown promising results in increasing renal response when added to standard of care therapy while using less cumulative glucocorticoids (GC) [13, 14]. The efficacy of anti-CD19 chimeric antigen receptor (CAR) T-cell therapy in cases with refractory SLE further highlights the critical pathogenic roles of B cells and their potential as therapeutic target [15].

Despite its efficacy in achieving immunological control, treatment with B-cell depletion is rarely continued into the maintenance phase after initial remission was achieved. A recent *post hoc* analysis of the NOBILITY trial (a phase II randomized controlled trial evaluating B-cell depletion with obinutuzumab plus standard therapies compared with standard therapies alone in active proliferative LN) showed significant attenuation in estimated glomerular filtration rate (eGFR) decline and reduction of LN flares in the obinutuzumab-treated arm, suggestive of the long-lasting effect of B-cell depletion which extends beyond the early “induction” phase [16]. Infrequent infusions also carry the advantage of addressing treatment non-adherence with oral regimen—one of the most important causes of treatment failure in LN [17]. Therefore, we hypothesize that continuous B-cell depletion with anti-CD20 agent may be a promising long-term treatment strategy for both induction and maintenance of remission of LN. Here we review our experience in using RTX-based continuous B-cell depletion for relapsing, refractory or severe LN. We sought to evaluate the relapse-free and overall renal survival, reduction of GC and oral IS, as well as adverse events associated with this approach.

## MATERIALS AND METHODS

### Study population

We performed a retrospective case series of patients with relapsing, refractory and severe LN at diagnosis who received long-term RTX therapy along with other IS between August 2008 and January 2023 at the Vasculitis and Glomerulonephritis Center at Massachusetts General Hospital. We defined long-term RTX therapy as administration of RTX for at least 12 months aiming for sustained B-cell depletion for induction and maintenance of remission, with a treatment goal of reducing GC and oral IS. Relapsing LN is defined as patients who experienced at least one renal relapse while receiving maintenance IS; refractory LN is defined as those who had at least one prior treatment non-responsiveness; severe LN was adjudicated by the treating physician, including patients with clinical presentation of rapidly progressive glomerulonephritis (RPGN) with histological features indicating high activity index (cellular crescents, fibrinoid necrosis), nephrotic syndrome with or without complications (e.g. acute thrombosis). Patients with LN who had concurrent refractory/severe extra-renal disease were also treated similarly as those with RPGN. Patients with non-SLE active autoimmune diseases were excluded. Patients were followed from the date of RTX initiation (time 0) to their last RTX infusion. Clinical outcomes after RTX discontinuation are described in patients with available follow-up data.

### Treatment regimen

The RTX dosing protocol was designed to induce and maintain continuous B-cell depletion. RTX was initially administered as two 1000 mg intravenous doses separated by 2–4 weeks, followed by one 1000 mg intravenous dose every 4 months [18]. Peripheral B cells were checked prior to each dose of maintenance RTX infusion, and if early B-cell repopulation (defined as presence of ≥5 peripheral B cells/µL) was found, the next dosing interval would be shortened to 3 months. Among patients who had achieved continuous B-cell depletion for >24 months and remained in remission, future dosing interval was gradually extended to allow B-cell repopulation to reduce risk of adverse events [19].

A combination of GC and oral IS [cyclophosphamide (CYC), mycophenolate mofetil/mycophenolic acid (MMF/MPA), azathioprine (AZA)] were administered with RTX in patients with active LN during induction IS, with an intention by the treating physicians that all oral IS be tapered off within 12 months after RTX initiation. The choice of concomitant IS was based on the individual’s prior treatment history and side effect profile, preference for avoiding reproductive side effects (i.e. CYC) and presence of active extra-renal disease.

### Study definition and outcomes

Complete remission (CR) was defined as a normal creatinine (or <15% rise in creatinine if abnormal at baseline) and urine protein to creatinine ratio (UP/Cr) <0.5 g/g. Partial remission (PR) was defined as a <15% rise in creatinine from baseline and at least a 50% reduction in UP/Cr to <1.0 g/g (if baseline UP/Cr was ≤3.0) or to UP/Cr ≤3.0 g/g (if baseline UP/Cr was >3.0). No response was defined as not having met criteria for CR or PR [11]. Renal relapse after remission is defined as doubling of UP/Cr from the nadir UP/Cr with or without increase in serum creatinine. Given the differences in the UP/Cr nadir achieved, an absolute UP/Cr ≥1 g/g is required for diagnosis of renal relapse in patients who have achieved CR, and UP/Cr ≥2 g/g in patients who have only achieved PR [20]. In LN with advanced chronicity, proteinuria-based criteria may not be sufficiently specific to differentiate between immunological activity and chronic damage, and a repeat biopsy was performed when clinically indicated and feasible to help determine renal relapse. End stage kidney disease (ESKD) was defined as requirement of kidney replacement therapy, including dialysis and transplant.

Additional study outcomes included time-to-remission, time-to-relapse, time-to-ESKD, longitudinal change in eGFR, UP/Cr and immunological markers, effect of oral IS agent reduction, extra-renal relapse and serious adverse events. Extra-renal relapse is defined as emergence of British Isles Lupus Assessment Group (BILAG) A score, which suggests active disease that required additional immunosuppressive drugs and/or prednisone dose of ≥20 mg daily. Adverse events including severe infection (infection requiring hospitalization), end-organ damage attributed to RTX therapy (non-infectious inflammatory conditions), neutropenia [defined as new-onset absolute neutrophil count (ANC) <1500 cells/mm^3^], hypogammaglobulinemia [defined as immunoglobulin G (IgG) persistently <400 mg/dL on at least on two measurements 4 months apart] were described.

### Statistical analysis

Median and interquartile range (IQR) were used to summarize continuous variables; frequencies and percentage were used to summarize categorical variables. Wilcoxon signed rank sum tests were used to compare longitudinal change of renal indices within a patient. Kaplan–Meier curves were used to describe time-to-remission and relapse-free survival. Log-rank test were used to compare two Kaplan–Meier curves. The study was approved by the Mass General Brigham institutional review board (2022P001037). Patient consent was waived for retrospective data collection.

## RESULTS

### Baseline characteristics

Twenty-six patients with LN were included (Table 1). Eighty-eight percent (*n* = 23) patients were female, 54% (*n* = 14) were White. Median age at RTX initiation was 35 years (IQR 25–43). Fifteen patients had class III or IV LN at initial kidney biopsy, eight had co-existing class III/IV and class V LN, and three had pure class V LN. Median duration of LN before RTX initiation was 4 months (IQR 0–31). Given limited number of male patients in our cohort (*n* = 3), sex-stratified analyses were not performed.

**Table 1:** Baseline characteristics of 26 patients treated with RTX for LN by individual.

Pt/sex age/ethnicity	LN/SLE duration (months)	Phenotype	LN class	Failed IS therapies	Cr (mg/dL)	UP/Cr (g/g)	Extra-renal LN
1/F 24/W	0/36	Severe initial (**RPGN**, >80% cellular crescents, severe CNS vasculopathy)	IV	HCQ	1.8	5.3	Ray, J, S, **CNS**
2/F 39/H	0/72	Severe initial (glomerular necrosis on biopsy, severe ILD)	IV	MTX, GC, LFU	0.8	0.64	**P**, Ray, J, S, He
3/F 33/W	0/0	Severe initial (**RPGN**: renal TMA)	IV	N/A	On dialysis	11.9	J, **TMA**
4/M 18/W	0/0	Severe initial (**RPGN**: 20% cellular crescents)	IV	N/A	1.71	8.2	None
5/F 43/B	2/12	Severe initial (class V with nephrotic syndrome)	V	N/A	0.66	3.1	**J**, Ray, E, O
6/F 49/A	0/192	Severe initial (class V with nephrotic syndrome)	V	GC, MMF, HCQ	0.72	2.61	Ray, S, He
7/F 47/B	0/115	Severe initial (+ severe recurrent myalgia, arthritis, cytopenia)	III	HCQ, belimumab	1.57	0.53	**J, He**, M
8/F 18/W	0/1	Severe initial (glomerular necrosis) on biopsy	IV + V	N/A	0.57	5.35	J, LAD, Sys
9/F 38/W	0/240	Severe initial (+pleuritis, pericarditis)	IV	GC, MMF	0.67	0.7	**Se**, He, E, J, L
10/M 68/W	0/0	Severe initial (**RPGN**: 60% crescents)	IV	N/A	On dialysis	4.59	None
11/F 53/W	1/1	Severe initial (**RPGN**: DPGN)	IV	GC, MMF, CYC	On dialysis	5.91	He
12/F 37/W	4/240	Refractory	III + V	GC, MMF	0.8	1.6	J, S, Ray, O
13/F 25/H	38/72	Refractory	IV + V	GC, MMF, belimumab	0.59	4.02	Se, S, J, Sys
14/M 35/B	4/4	Refractory	V	GC, MMF	1.03	2.5	**S, J, Se**
15/F 24/H	36/108	Refractory	IV + V	GC, MMF	0.99	4.84	Ray, J
16/F 41/W	1/3	Refractory	III + V	GC, MMF	0.94	7	**APLA, Sys**
17/F 25/W	11/24	Relapsing	IV	GC, MMF, MTX	1.0	3.0	J, **S**
18/F 35/W	105/156	Relapsing	IV + V	GC, MMF, AZA, CYP	2.74	4.0	J
19/F 43/H	119/132	Relapsing	III	GC, MMF, belimumab, AZA, MTX	0.64	1.58	J, S, He
20/F 31/H	43/108	Relapsing	III + V	GC, MMF	1.19	1.49	J, S, O
21/F 23/W	6/84	Relapsing	IV	GC, MMF, TAC	1.15	0.86	J, M, S
22/F 22/H	11/24	Relapsing	IV	GC, MMF	0.61	4.05	J, S, Se
23/F 58/W	147/156	Relapsing	IV	GC, MMF, CYC	2.9	5.89	J
24/F 33/A	14/99	Relapsing	IV	GC, MMF, belimumab, abatacept, tofacitinib	1.06	4.63	S, J, He
25/F 41/W	2/22	Relapsing	III + V	GC, MMF	0.66	2.38	S
26/F 21/H	40/48	Relapsing	IV	GC, MMF, CYC	2.7	12.78	CNS, Se, M

Baseline characteristics by individual patient. Serum creatinine (Cr) and UP/Cr were collected from the most proximal value prior to RTX initiation. Pt, patient; B, Black; W, White; H, Hispanic; A, Asian; DPGN, diffuse proliferative glomerulonephritis; TMA, thrombotic microangiopathy; MTX, methotrexate; LFU, leflunomide; HCQ, hydroxychloroquine. Extra-renal manifestations shown in bold indicates active disease at the time of RTX initiation. S, cutaneous manifestation (rash, alopecia, etc.); J, arthritis/arthralgia; Ray, Raynaud’s phenomenon; Se, serositis (pericarditis, pleuritis); He, hematological manifestations (cytopenia, autoimmune hemolytic anemia, TMA, etc.); CNS, central nervous system involvement (CNS vasculopathy, cerebritis, posterior reversible encephalopathy syndrome); LAD, lymphadenopathy; E, eye involvement (uveitis, scleritis, etc.); O, oral involvement (oral ulcer, sicca syndrome, etc.); P, pulmonary involvement (ILD, pulmonary arterial hypertension, etc.); ILD, interstitial lung disease; APLA, antiphospholipid syndrome; M, myalgia/myositis; L, liver involvement (hepatitis, etc.).

RTX was initiated for induction of remission and continued for maintenance of remission in all 26 patients. Thirty-eight percent (*n* = 10) patients had relapsing LN, 19% (*n* = 5) patients had refractory LN and 42% (*n* = 11) patients had LN with severe features at diagnosis, including RPGN (*n* = 5), LN with concurrent refractory/severe extra-renal disease (*n* = 4) and class V LN with severe nephrotic syndrome (*n* = 2) (Table 1). At RTX initiation, three patients with RPGN were on dialysis (Patients 3, 10, 11) and three patients had advanced chronic kidney disease (CKD) with baseline serum creatinine >2 mg/dL (Patients 18, 23, 26).

### B-cell depletion using RTX

The median duration of RTX therapy was 32 months (IQR 14–68), and the median cumulative dose was 10 g (IQR 6–17). During the first 12 months of RTX treatment, 31% (8/26) of patients experienced early B-cell repopulation necessitating shortening RTX dosing interval to 2–3 months. With dosing interval adjustment, continuous B-cell depletion was achieved in 92% (23/25) patients at 12 months (excluding Patient 24 who was switched to ocrelizumab after developing serum sickness with RTX infusion). At 24 months, B-cell depletion was maintained in 88% (14/16) patients, except for Patients 14 and 22 who continued to have early B-cell repopulation despite shortening dosing interval. Of the 15 patients who received RTX for >24 months, maintenance RTX dosing interval was gradually extended in 11 patients to allow partial B-cell repopulation. For the remaining four patients, RTX was continued every 4 months due to active renal/extra-renal symptoms (*n* = 2) or advanced CKD with limited renal reserve to tolerate risk of renal relapse (*n* = 2) (Table 2). We did not identify any baseline characteristics (proteinuria, C3/C4 levels, disease vintage, etc.) associated with early B-cell repopulation in our cohort (data not shown).

**Table 2:** Immunosuppressive regimens and outcomes by individual.

Pt	Initial Pred (mg/day)^a^	Other meds (duration, months)	RTX doses/duration (months)	Remission (months)	Renal relapse (months)/B cells	Extrarenal relapse (months )	RTX extended after 24 months	Other IS at last RTX	Follow-up after last RTX (months)	Cr at last follow-up (mg/dL)
1/F 24/W	80	CYC (2), HCQ	20/78^a^	CR (6)	Yes (70)/B cell +		Yes	0	0	0.74
2/F 39/H	60	CYC (2), MMF (9), HCQ	5/12	CR (1)	None			MMF, HCQ	45	0.88
3/F 33/W	40	CYC (2)	7/38	PR (4)	None		No (advanced CKD)	0	15	ESKD at 53 months
4/M 18/W	40	CYC (2)	11/50	CR (4)	None		Yes	0	0	0.98
5/F 43/B	30	CYC (2)	8/24	PR (11), CR (24)	None			0	22	0.68
6/F 49/A	30	CsA (2), CYC (2)	17/68^a^	PR (3), CR (24)	None		Yes	0	0	0.8
7/F 47/B	60	CYC (2), MPA (4)	5/13	None	N/A			0	0	2.05
8/F 18/W	60	CYC (2), MMF (4)	6/19	CR (1)	None			0	0	0.76
9/F 38/W	30	CYC (2), AZA (1), MMF (14), HCQ	7/23	CR (4)	None	Pericarditis, pleuritis (6, 13)/B cell –		Rilonacept	0	0.70
10/M 68/W	60	CYC (2), HCQ	5/12	None	N/A			0	0	ESKD at 12 months
11/F 53/W	30	CYC (2)	13/67	CR (3)	None		Yes	0	0	0.74
12/F 37/W	20	CYC (3), MMF (34), HCQ	11/42^a^	PR (23)	None	Dermatitis (15)/B cell –	Yes	MMF	40	0.84
13/F 25/H	20	MMF (6), CYC (2), MTX (36)	18/80	CR (2)	None	Pleuritis, pericarditis, dermatitis (multiple)/B cell –	No (active extra renal symptoms)	MMF	22	0.52
14/M 35/B	30	CYC (2), MMF (12)	27/104^a^	None	N/A	Arthritis, dermatitis (65)/B cell +	Yes	0	0	1.49
15/F 24/H	15	HCQ (13)	5/13	CR (9)	None			0	0	0.81
16/F 41/W	40	CYC (2), HCQ	6/14^a^	PR (4)	N/A			0	0	0.72
17/F 25/W	30	CYC (3), MMF (10)	15/81	CR (4)	None		Yes	0	34	0.72
18/F 35/W	20	CYC (2), MMF (5)	11/35	PR (8)	Yes (36)/B cell –		No (advanced CKD)	0	2	ESKD at 37 months
19/F 43/H	20	MMF (1), HCQ	6/15	PR (12)	None	Dermatitis (7)/B cell –		0	51	0.71
20/F 31/H	15	MPA (82)	27/162	CR (4)	Yes (142)/B cell +	Arthritis, myalgia, alopecia (53)/B cell –	Yes	0	0	0.91
21/F 23/W	60	MMF (2), TAC (2), CYC (2), HCQ	12/28^a^	None	N/A	Pleuritis (6)/B cell +	Yes	0	63	2.13
22/F 22/H	15	MMF (53), HCQ	20/58^a^	PR (4)	Yes (24)/B cell +		No (active LN, refractory to B-cell depletion)	MMF, voclosporin	0	0.51
23/F 58/W	30	CYC (6)	20/101	PR (8)	Yes (43, 78)/B cell +		Yes	0	7	ESKD at 108 months
24/F 33/A	15	CYC (1), MMF (1), AZA (12)	5/14	PR (2)	None			AZA	0	0.94
25/F 41/W	20	AZA (10), HCQ	7/25	CR (8)	None		Yes	0	0	1.01
26/F 21/H	60	CYC (1), MMF (9)	7/13^a^	None	N/A			MMF	1	ESKD at 14 months

Treatment regimen, B-cell status and clinical outcomes. For patients who received methylprednisolone, dose was converted to daily prednisone equivalent. ^a^Indicates patients who experienced early B-cell repopulation during the first 12 months of RTX treatment. Patient 24 experienced serum sickness with first RTX infusion and was subsequently switched to ocrelizumab. In our protocol, oral CYC was administered at 2 mg/kg daily for 1 week, then 1 mg/kg daily for 7 weeks with the following adjustments for eGFR (10% dose reduction if eGFR was 60–90 mL/min/1.73 m^2^, 25% if 45–59 mL/min/1.73 m^2^, 33% if 30–44 mL/min/1.73 m^2^, 40% if 15–29 mL/min/1.73 m^2^ and 50% if <15 mL/min/1.73 m^2^ or on dialysis). In several patients with persistent disease activity, oral CYC course was extended to 3 months (Patient 23 received total 6 months of CYC). Among all patients who were exposed to CYC (*n* = 21), median cumulative dose was 4875 (IQR 4300–6188) mg. Pt, patient; B, Black; W, White; H, Hispanic; A, Asian; CsA, cyclosporine;TAC, tacrolimus.

### Induction of remission in active LN

Eighty-one percent (21/26) patients achieved at least PR during follow-up (CR = 11, PR = 10). Median time-to-remission was 4 months (IQR 3.5–8.5). The majority of patients (73%, 19/26) received short course (2–3 months) of oral CYC in addition to rapid prednisone taper at the time of RTX initiation, while others received MMF/MPA (*n* = 4), MMF + TAC (*n* = 1) or AZA (*n* = 1), and one patient did not receive additional oral IS. There was no significant difference in time-to-remission between patients with severe LN at diagnosis and those with relapsing/refractory LN, and between those who received CYC and non-CYC based regimen for induction of remission (Supplementary data, Fig. S1).

### Relapse-free survival

**Figure 1:**
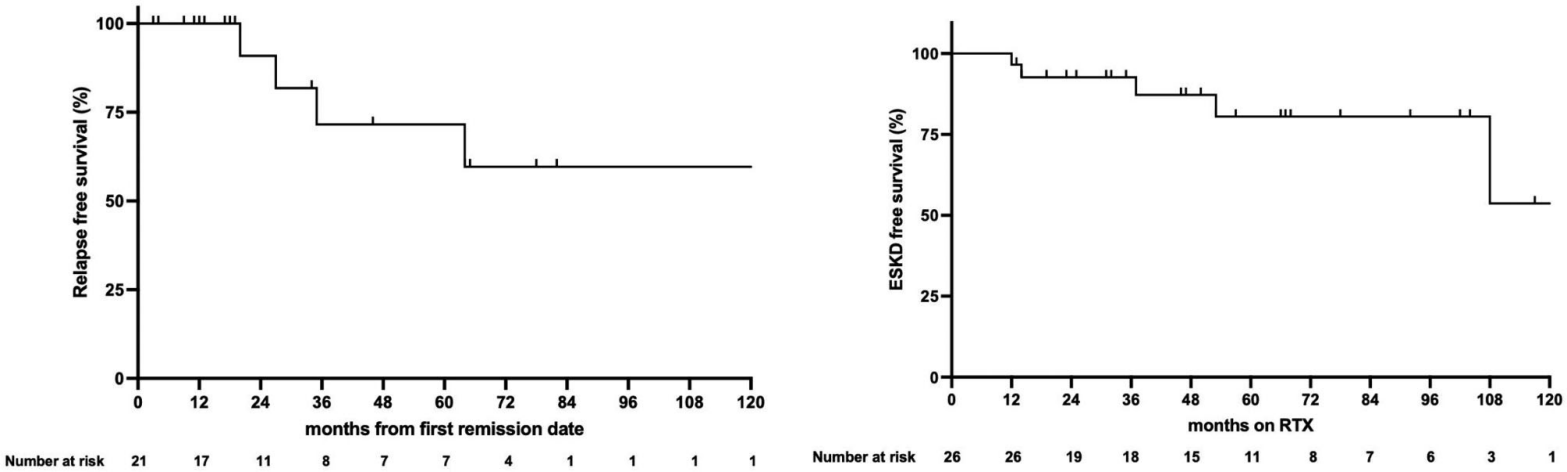
Relapse-free survival and ESKD-free survival. (**A**) Relapse-free survival among patients who achieved remission (*n* = 21) and (**B**) ESKD-free survival of the entire cohort (*n* = 26). ESKD status was declared at 12 months in Patient 10 who presented with RPGN requiring dialysis and failed to achieve dialysis independence.

Among the 21 patients who achieved at least PR, 6 episodes of renal relapse occurred in 5 patients. Median time-to-first relapse was 43 months (range 24–142). Relapse-free survival was 100% at 12 months, 91% at 24 months and 72% at 36 months (Fig. 1A). Five of the six episodes of renal relapses in our cohort were preceded by B-cell repopulation, and a detailed description of the relapse episodes is shown in Table 3.

**Table 3:** Description of renal relapses.

Pt	UP/Cr at end of induction (g/g)	Cr at end of induction (mg/dL)	Time on RTX therapy (months)	B-cell depleted at the time of relapse	UP/Cr at relatoppse	Cr at rppelapse (mg/dL)	IS at relapse	Course/treatment
1	0.18	0.81	70	No (previous RTX 8 months)	1.06	0.74	RTX (q 8 months)	Reinduction of complete B-cell depletion with RTX (1 g, 2 weeks apart), followed by continuous B-cell depletion for 12 months; not yet in remission at last follow-up (78 months)
21	1.05	2.58	35	Yes	2.82	3.45	RTX (q 4 months)	Difficult to distinguish renal relapse vs CKD progression; given advanced CKD at RTX initiation and marginal benefit with additional intensive IS, patient received preemptive renal transplant at 37 months
23	0.28	0.9	142	No (previous RTX 8 months)	2.08	0.99	RTX (q 8 months)	Continued RTX with shortening of dosing interval (6 months); UP/Cr improved to 0.5 g/g at 145 months
25	1.12	0.6	24	No (previous RTX 4 months)	2.74	0.58	RTX (q 4 months) + MMF 2000 mg daily	Complete B-cell depletion not achieved with RTX q 3-month dosing; UP/Cr worsening; voclosporin was added at last follow-up
26	1.95	2.8	43	No (previous RTX 10 months)	6.48	2.3	RTX (q 10 months)	Reinduction of complete B-cell depletion with RTX (1 g, 2 weeks apart) and two doses of IV methylprednisolone. UP/Cr improved to 1.87 g/g at 44 months
26	1.95	2.8	78	No (previous RTX 10 months)	4.57	2.3	RTX (q 10 months)	Reinduction of complete B-cell depletion with RTX (1g, 2 weeks apart) and two doses of IV methylprednisolone; UP/Cr improved to 1.99 g/g at 85 months

Description of each relapse episode with associated treatment and outcome. Pt, patient; q, every; IV, intravenous.

### ESKD-free survival

ESKD occurred in five patients. Median time-to-ESKD was 37 months (range 12–108). ESKD-free survival was 97% at 12 months, 92% at 24 months and 92% at 36 months (Fig. 1B). Two patients were on dialysis at RTX initiation: Patient 10 remained dialysis-dependent at 12 months despite IS, and Patient 3 was liberated from dialysis initially but subsequently developed worsening of kidney function and was declared to have ESKD at 53 months. The remaining three patients (Patients 18, 23, 26) all had baseline Cr >2 mg/dL with advanced chronic changes on biopsy at RTX initiation.

### Reduction in oral IS

**Figure 2:**
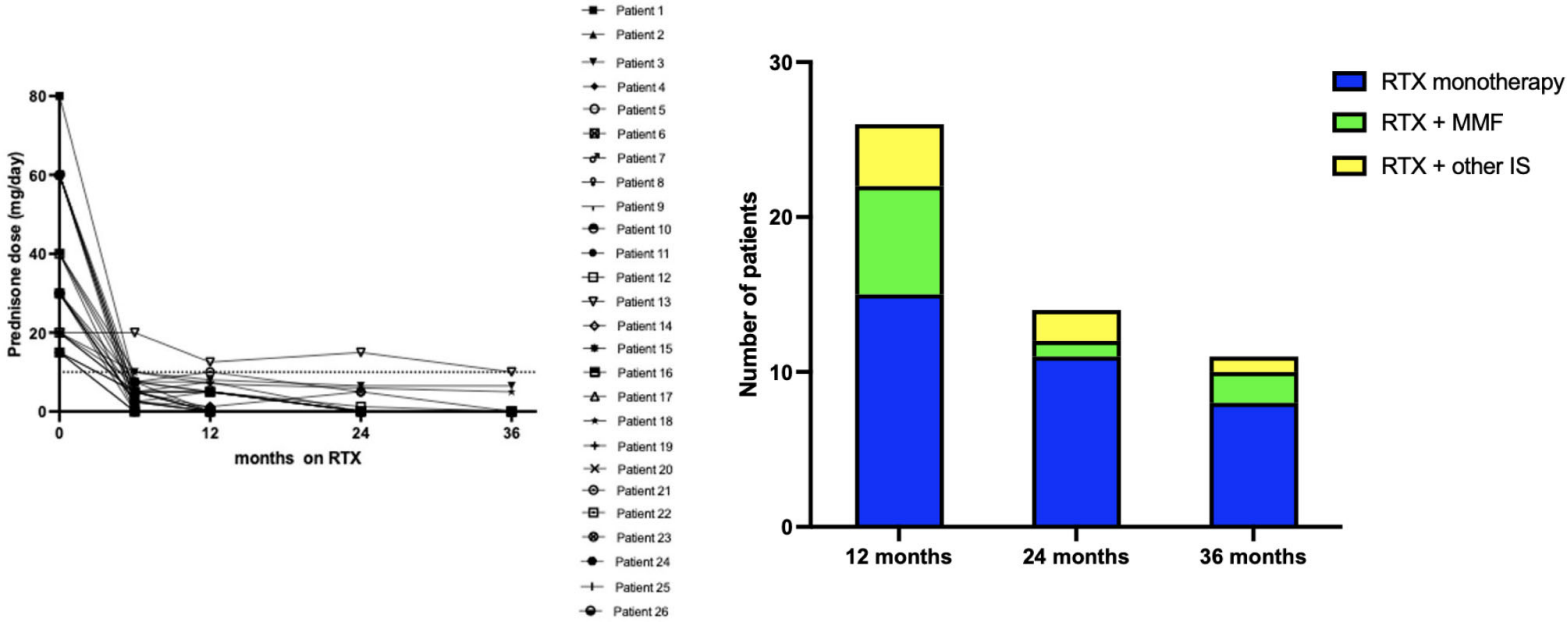
Reduction in prednisone dose and additional IS. Reduction in daily prednisone dose (**A**) and additional IS (**B**). Other IS includes AZA, prednisone (>7.5 mg/day) and rilonacept (one patient).

The median prednisone dose decreased from 30 mg/day (IQR 20–55) at RTX initiation to 5 mg/day (IQR 2.5–7.5) at 6 months and 1 mg/day (IQR 0–5) at 12 months (*P* < .01) (Fig. 2A). Only one patient (Patient 13) with refractory extra-renal symptoms (arthritis, pleuritis) remained on >10 mg/day of prednisone at 12 months. Fifteen of 26 (58%) patients were receiving RTX monotherapy at 12 months, 11/14 (79%) at 24 months and 8/11 (73%) at 36 months (Fig. 2B). Details of patients who required additional concurrent IS are described in Table 2.

### Longitudinal change in renal indices and immunological markers

**Figure 3:**
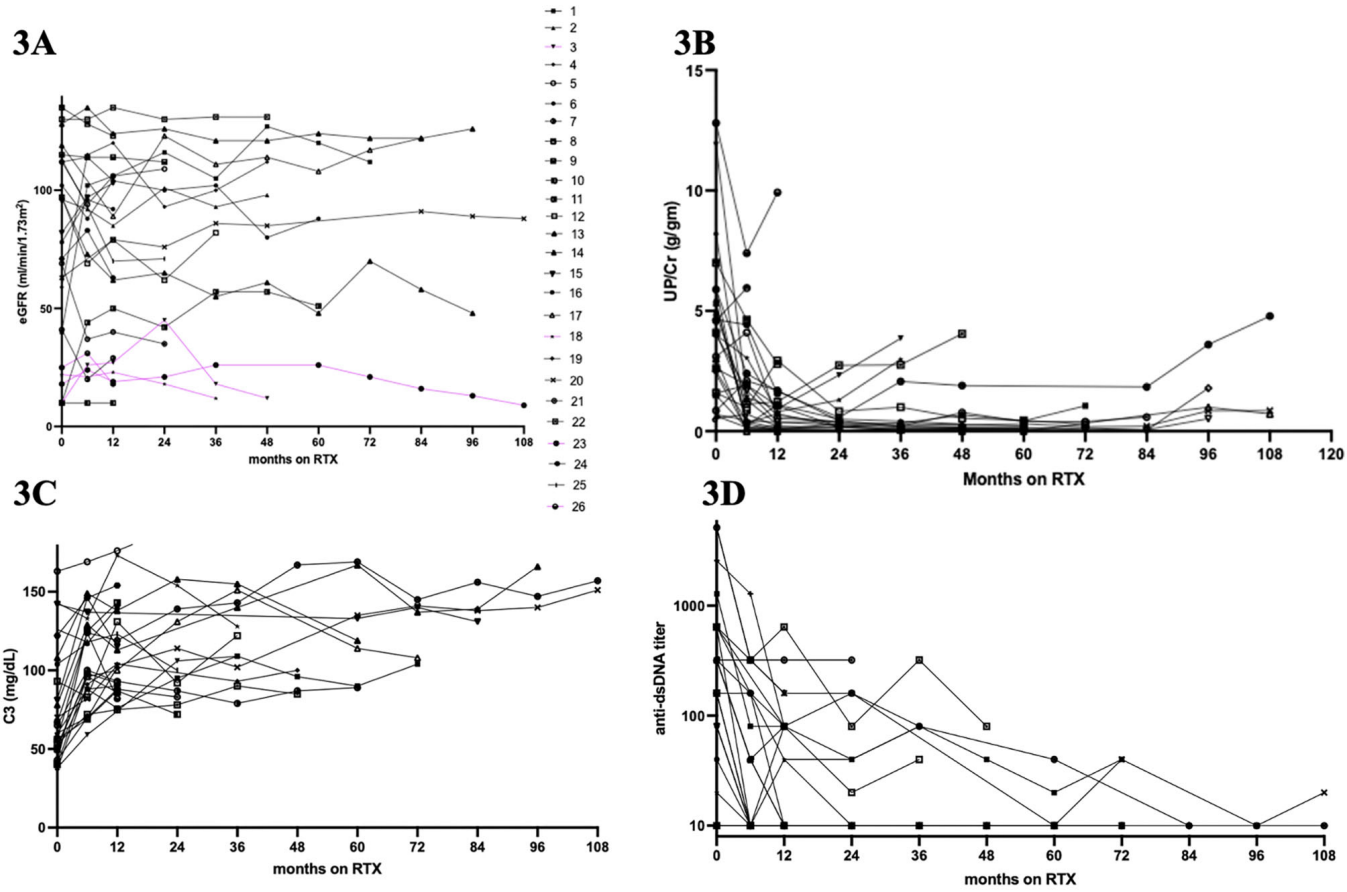
Longitudinal change of biomarkers. Longitudinal change in eGFR (**A**), UP/Cr (**B**), C3 (**C**) and anti-dsDNA titer (**D**) from RTX initiation. Pink connecting line in (A) indicates patients who developed ESKD during follow-up (Patients 3, 18, 23, 26). Patient 10 presented with RPGN requiring dialysis and did not liberate from dialysis (eGFR was imputed as 10 mL/min/1.73m^2^ on the plot).

Longitudinal changes in eGFR, UP/Cr, C3 and dsDNA levels are shown in Fig. 3A–D. Significant improvement in UP/Cr, C3 and anti-dsDNA titers were observed at 12 months compared with baseline: median UP/Cr decreased from 4.0 g/g (IQR 1.8–5.3) to 0.6 g/g (IQR 0.2–1.2) (*P* < .01); median C3 increased from 63 (IQR 51–101) to 103 (IQR 89–136) (*P* < .01); and median anti-dsDNA titer decreased from 1:160 to 1:10 (*P* < .01).

### Renal outcome in patients with RPGN

Five patients presented with RPGN, and three required dialysis at RTX initiation (Patients 3, 10, 11). All five patients received a short course of combination oral CYC/GC at RTX initiation. Patients 3 and 11 were liberated from dialysis at 4 months and 1 month, respectively. Patient 10 remained dialysis-dependent and underwent kidney transplantation at 12 months. Patients 1, 4 and 11 all continued RTX monotherapy as maintenance, and remained relapse-free with stable renal function until last follow-up (78, 50 and 74 months, respectively) (Table 2).

### Extra-renal relapse

Seven patient developed extra-renal relapse requiring addition of prednisone ≥20 mg daily and/or additional IS. Most common extra-renal relapses include rash/dermatitis (*n* = 4), pleuritis (*n* = 3) and pericarditis (*n* = 2). Five out of the seven episodes occurred during complete B-cell depletion. None of the extra-renal relapse occurred with concurrent renal relapse (Table 2).

### RTX discontinuation

Forty-six percent (12/26) of patients discontinued RTX therapy during follow-up (Table 2). Reasons for RTX discontinuation include ESKD (Patients 3, 10, 18, 23, 26), pregnancy (Patients 17, 21), infections (Patients 5, 12), socioeconomic factors (Patients 2, 19) and inadequate control of extra-renal symptoms (Patient 13). Patients 17 and 21 both restarted RTX monotherapy as maintenance IS after successful, uncomplicated pregnancies.

### Adverse events

Total time of exposure to RTX therapy was 1188 patient-months. No deaths occurred during follow-up. RTX infusions were generally well-tolerated, except for one (Patient 24) who developed serum sickness after the first RTX infusion and subsequently switched to ocrelizumab. Twelve episodes of severe infections occurred in 31% patients (10 events per 1000 patient-months), described in Supplementary data, Table S2. Pyelonephritis and diverticulitis were the most common infections that led to hospitalization, and most events occurred during the early induction phase of IS while on CYC and prednisone (Patient 3 developed hip septic arthritis, and Patient 5 developed CMV viremia). During prolonged B-cell depletion (>24 months), three patients developed chronic bronchitis and two patients developed inflammatory vaginitis—all improved with holding/extending RTX dosing interval to allow partial B-cell repopulation (Supplementary data, Table S2). Neutropenia occurred in 24% (6 events per 1000 patient-months): four were attributed to MMF, one to CYC, one to lupus activity and one to RTX. Patient 23 developed severe late-onset neutropenia attributed to RTX (ANC nadir 70 cells/mm^3^) requiring temporary filgrastim; RTX was later resumed at an extended interval without recurrence of neutropenia. Hypogammaglobulinemia (defined by IgG <400 mg/dL) occurred in three patients (3 events per 1000 patient-months), including Patient 3 who developed severe hypogammaglobulinemia (IgG <40 mg/dL) requiring monthly intravenous immunoglobulin support. The patient had been on RTX for 38 months and did not achieve B-cell repopulation despite discontinuing RTX for >24 months. The other two patients did not develop severe infection during follow-up, and one patient had improvement in IgG levels to >400 mg/dL after withholding RTX (Patient 26), while no repeat IgG level was available for the other (Patient 5). Nine patients had at least one episode of COVID-19 infection during follow-up: all were treated with supportive care with or without antiviral therapy, and none required hospitalization.

## DISCUSSION

In our retrospective case series of patients with LN treated with long-term RTX with an intention of sparing oral IS, we observed a 2-year relapse-free survival of 91% among patients presenting with relapsing, refractory or severe LN who achieved remission. The reduction in GC was significant (88% patients reduced prednisone dose to ≤7.5 mg/day by 6 months) and over half of patients were able to discontinue oral IS and maintain remission on RTX monotherapy after 1 year. Most patients showed early and sustained improvements in biomarkers (complements, proteinuria, anti-dsDNA), consistent with trials of RTX and obinutuzumab [11, 14]. Compared with other case series assessing RTX as induction IS in relapsing, refractory or severe LN, our study uniquely evaluates the long-term efficacy and tolerability of RTX, as well as its potential as single maintenance agent in patients with prior treatment failure or advanced chronicity at diagnosis [21–23]. We also evaluated long-term clinical outcomes in patients with RPGN or advanced CKD who are typically excluded in clinical trials.

In addition, our study showed a much faster rate of B-cell repopulation in patients with LN (including two patients who failed to achieve continuous B-cell depletion despite increase in frequency of RTX dosing) compared with other autoimmune glomerulonephritis (e.g. ANCA vasculitis, membranous nephropathy) when using the same dosing protocol (2× 1000 mg induction, then 1000 mg every 4 months) [24, 25]. This aligns with other studies and may be associated with multiple mechanisms including higher target latent antigen burden in LN, impaired complement-mediated cytotoxicity, elevated B-cell activating factor (BAFF) and development of anti-drug antibodies (ADA), etc. [24–27]. The formation of ADA to RTX is common in patients with SLE and may be particularly important in contributing to “RTX resistance” [26, 28]. It is possible that shortening the starting intervals of RTX (e.g. employing 375 mg/m^2^ weekly dose ×4) may prevent the formations of ADA through “desensitization” and offer persistent excess of neutralizing antibody. Additionally, guiding the maintenance dosing interval with frequent monitoring of peripheral CD19 counts (e.g. monthly check instead of every 4 months) would allow more pre-emptive depletion of repopulated autoreactive B cells. The optimal RTX dosing regimen that offers most sustained B-cell depletion in LN remains unknown and requires further study.

Repopulation of B cells preceded majority of the renal relapses in our cohort, but most extra-renal flares (serositis, dermatitis) occurred despite complete B-cell depletion. This underscores the inherent heterogeneity in organ-specific pathogenesis in SLE and suggests that a more comprehensive assessment of the immunophenotype may be helpful in predicting risk of relapse [5, 29]. Future studies evaluating long-term RTX therapy with both renal and extra-renal endpoints would be needed to evaluate continuous B-cell depletion as the therapeutic approach to maintain global autoimmune quiescence in SLE.

Approximately one-third of patients with LN in our cohort developed at least one episode of serious infection, comparable to a recent study showing serious infection rate of 34% among patients with various autoimmune diseases treated with long-term RTX therapy (median time on RTX was 54 months) [30]. Most of the severe infections occurred during the early induction phase when concurrent oral IS was used, and it is difficult to discern the individual contribution of RTX. Nineteen percent (5/26) of patients developed chronic bronchitis or desquamative inflammatory vaginitis—two chronic inflammatory conditions associated with prolonged B-cell depletion and immune dysregulation [31]. Although most patients experienced symptomatic improvement with B-cell repopulation, these conditions could lead to significant impairment of quality of life and serve as disadvantages for long-term RTX therapy. Late-onset neutropenia and hypogammaglobulinemia were largely reversible with holding RTX; however, one patient did develop severe hypogammaglobulinemia with failure to achieve B-cell repopulation, and it remains an important consideration of long-term RTX therapy.

Overall, despite the emergence of newer therapies like obinutuzumab and CAR T-cell therapy which achieve more potent B-cell depletion, we propose that the use of RTX should not be completely abolished, especially given the lower accessibility and higher cost of type II anti-CD20 agents and cell therapies. Our study supports that RTX still may have a role in treatment of LN by being repurposed as a long-term agent through inducing continuous B-cell depletion, as opposed to the traditional opinion of using it as “induction” therapy with fixed dosages and intervals only in patients with acute disease. Furthermore, although our study only included patients with relapsing/refractory LN or those with severe initial manifestation, this treatment approach may also be evaluated in patients with non-severe, newly diagnosed LN to achieve early treatment success. Future randomized controlled trials evaluating different dosing regimens of anti-CD20 agents as the backbone therapy would offer additional insights into “continuous B-cell depletion” as a key concept in maintaining autoimmune quiescence of LN.

Our study has several limitations. It is a retrospective, single-center study and lacks a comparison group to evaluate the comparative efficacy of RTX. However, most patients in our cohort had history of multiple treatment failures with limited options of an alternative agent. Additionally, although our goal was to evaluate the efficacy of continuous B-cell depletion using RTX as a single agent for treatment of LN, it was not feasible in a subset of patients with active extra-renal disease which drove the treatment with IS. This further highlights the complexity of treatment of SLE and suggests that future studies using different endpoints are needed to evaluate the efficacy of RTX monotherapy in controlling global disease activity of SLE. Finally, our study did not measure repopulation pattern of B-cell subsets (e.g. memory B cells, plasmablasts), which may serve as more sensitive biomarkers for reconstitution of autoreactive B cells and prediction of relapse.

## CONCLUSIONS

Long-term continuous B-cell depletion with RTX demonstrates a high rate of relapse-free survival with favorable steroid-sparing effect and oral IS reduction in patients with relapsing, refractory or severe LN.

## Supplementary Material

sfag152_Supplemental_File

## Data Availability

QW reports funding by National Kidney Foundation (GR0131514) and GlomCon Physician Scientist Scholarship (GR0131625).
