# Supplementary material for: Long-term B-cell depletion with rituximab in relapsing, refractory and severe lupus nephritis: a retrospective case series
Source: Clin Kidney J. 2026 May 11;19(6):sfag152. doi: 10.1093/ckj/sfag152 (PMC13224835; doi:10.1093/ckj/sfag152)
Supplement: sfag152_Supplemental_File [file sfag152_supplemental_file.docx]

**Supplemental materials**

**Table S1. Repeat renal biopsies during follow-up-----------------------------------------------------------------------------------------------------------------7**

**Table S2. Adverse events----------------------------------------------------------------------------------------------------------------------------------------------8-9**

**Figure S1. Time-to-remission among patients with active LN at RTX initiation--------------------------------------------------------------------------10**

**Supplemental Table 1. Repeat kidney biopsies during follow-up**

| **Patient** | **Time of repeat biopsy**  **(mos)** | **UP/Cr at repeat biopsy (g/g)** | **Cr at repeat biopsy (mg/dL)** | **Biopsy findings** |
| --- | --- | --- | --- | --- |
| 3 | 43 | 2.6 | 3.8 | Advanced chronicity (65% global glomerulosclerosis, chronic and active interstitial nephritis) |
| 20 | 91 | 0.74 | 2.07 | Advanced chronicity (75% global glomerulosclerosis, 60% IFTA, moderate-severe arteriosclerosis) |
| 26 | 12 | 9.92 | 3.95 | Advanced chronicity (69% global glomerulosclerosis, 70% IFTA, moderate arteriosclerosis) |

**Table S1**. Description of the biopsy findings among patients who underwent repeat kidney biopsy during follow-up. mos = months, UP/Cr = urine protein to creatinine ratio, IFTA = interstitial fibrosis and tubular atrophy.

**Supplemental Table 2. Adverse events**

| **Patient** | **Severe infections/time of onset from RTX initiation (months)** | **Additional IS at the time of infection** | **End-organ damage associated with RTX /time of onset from RTX initiation (months)** | **other AEs/time of onset from RTX initiation (months)** |
| --- | --- | --- | --- | --- |
| 3 | Septic arthritis (2) | GC/CYC |  | Severe hypogammaglobulinemia (37)   - IgG < 40 mg/dL, requiring monthly IVIG support) |
| 5 | Systemic CMV (colitis, pneumonia) (2) | CYC |  | Hypogammaglobulinemia (6)   - nadir IgG 359 mg/dL) - IgG 379 at 16 months after last RTX infusion - No clinical obvious infection; did not receive IVIG |
| 6 |  |  | Chronic bronchitis (30) |  |
| 7 |  |  |  | Neutropenia (5)   - attributed to MFA, nadir ANC 600 cell/mm^3^ |
| 9 |  |  |  | Neutropenia (4)   - attributed to MMF, nadir ANC 1290 cell/mm^3^ |
| 11 | Recurrent diverticulitis (11, 36) | No |  |  |
| 12 | Pyelonephritis (46) | MMF | Inflammatory vaginitis (43) | Neutropenia (56)   - attributed to MMF, nadir ANC 1250 cell/mm^3^ |
| 13 |  |  | Inflammatory vaginitis (84) |  |
| 14 |  |  |  | Neutropenia (7)   - attributed to lupus activity, nadir ANC 540 cell/mm^3^ - improved with intensified immunosuppression |
| 20 | Recurrent pyelonephritis (117,128) | No | Chronic bronchitis (96) | Neutropenia (159)   - attributed to RTX LON, nadir ANC 70 cell/mm^3^ - required G-CSF support |
| 21 | Pyelonephritis (2) | CYC |  | Neutropenia (2)   - attributed to CYC, nadir ANC 830 cell/mm^3^ |
| 23 | Recurrent diverticulitis (78, 96) | No |  | Hypogammaglobulinemia (42)   - nadir IgG 244 mg/dL at 98 months - improved to 321 at last follow-up |
| 24 |  |  |  | Rituximab-induced serum sickness (1) |
| 25 | Acute Lyme carditis (32) | No |  |  |
| 26 |  |  |  | Neutropenia (14)   - attributed to MMF, nadir ANC 860 cell/mm^3^ |

**Supplemental Table 2.** Severe infection is defined as infection that is life-threatening or requiring hospitalization. End-organ damage attributed to RTX therapy is defined as non-infectious inflammatory condition that significantly impairs organ function or causes persistent symptoms. neutropenia is defined as new-onset absolute neutrophil count (ANC) < 1500 cell/mm3, hypogammaglobulinemia is defined as IgG persistently < 400 mg/dL on at least two measurements 4 months apart. abbreviations: MMF = Mycophenolate mofetil, CYC = cyclophosphamide, GC = corticosteroid, LON = late onset neutropenia

**Supplemental Figure 1. Time-to-remission among patients with active LN at RTX initiation**

**S1A S1B**

**Figure S1.** Time-to-remission among patients with active LN (n = 26) at RTX initiation by **(A)** disease phenotype (severe initial LN vs. relapsing/refractory disease) and **(B)** additional induction immunosuppression at RTX initiation (CYC-based vs. non-CYC based). No significant difference was observed between patients with severe initial LN and relapsing/refractory LN (*P* = 0.29, Log-rank test) and between patients received CYC as induction immunosuppression and those who received alternative agents (mycophenolate, azathioprine, etc) (*P* = 0.88, Log-rank test). One patient (patient 13) who were treated with MMF and one patient with MMF + TAC (patient 21) at RTX initiation later received short course of CYC during the induction period given persistent disease activity. LN = lupus nephritis; CR = complete remission; PR = partial remission
